# Case Report: A Painful Twist of Fate Due to Intra-abdominal Textiloma

**DOI:** 10.12688/f1000research.169895.1

**Published:** 2025-09-19

**Authors:** Rakia Siala, Mohamed Ali Mseddi, Haythem Yacoub, EYA AZOUZ, Hajer Hassine, Chaima Yacoubi, Meriem Hsairi, Fatma Trifa, Radhia Boubaker, Nesrine Krifa, Sallemi Karim, Emna Khemakhem, Abdelwaheb Mghirbi, Amal Bhira, Yosra Yahia, Souhir Mestiri, Rami Guizani, Brahim Ghariani, Karim Sassi, Hamida Maghraoui, Hela Kchir, Mohamed Ben Slima

**Affiliations:** 1 General surgery “B” department, Rabta Hospital, Tunis, Tunis, Tunisia; 2 Gastroenterology “B” Department, Rabta Hospital, Tunis, Tunis, Tunisia; 3 Medical Imaging Department, Rabta Hospital, Tunis, Tunis, Tunisia; 4 Emergency department, Rabta Hospital, Tunis, Tunis, Tunisia

**Keywords:** Textiloma, intestinal perforation, peritonitis

## Abstract

**Introduction:**

Textiloma refers to a retained surgical textile material within the body after surgery. It is a rare but serious iatrogenic complication that may remain asymptomatic for years.

**Case Presentation:**

We report a case of a 38-year-old woman with a history of open ovarian cystectomy 15 years earlier, presenting with persistent abdominal pain. Imaging revealed a complex pelvic mass with abscess formation and enteric fistula. Despite interventional drainage and antibiotic therapy, the patient developed signs of sepsis. Endoscopic evaluation revealed a retained surgical textile in the rectum, which was extracted. The patient subsequently developed generalized peritonitis and underwent emergency laparotomy revealing two retained textilomas and multiple intestinal perforations. Despite aggressive surgical intervention, the patient succumbed to septic shock on postoperative day one.

**Conclusion:**

Textiloma should be considered in patients with atypical abdominal masses and previous surgical history. Preventive strategies and early recognition are critical to avoid fatal outcomes.

## Introduction

Despite standardized operative protocols and heightened vigilance regarding surgical sponge counts, retained surgical items, particularly textiloma, continue to occur. Alarmingly, this complication persists even in high-resource healthcare settings. In Canada, surgical wards have reported that retained surgical items account for approximately 12% to 18% of surgical complications. ^1^ Whereas English authorities reported that gossypibomas represent 21% of never events. ^2^ It is a fearsome complication as it is often labeled as medical negligence ^3^ and its consequences might cost the patient his life and the surgeon his professional reputation. ^4^

## Case-report

A 38-year-old nulliparous woman with a history of open ovarian cystectomy 15 years prior was referred to our department for evaluation of abdominal pain evolving over one month, with nocturnal predominance. She denied smoking, alcohol use, or any known allergies. On admission, her vital signs were stable. Clinical examination revealed a patient with altered general condition (ECOG Performance Status = 2). Abdominal examination noted a healed sub-umbilical median laparotomy scar. No palpable mass, tenderness, or peritoneal signs were present.

Laboratory studies showed elevated C-reactive protein (CRP) at 80.5 mg/L and hypokalemia (K ^+^ = 2.7 mmol/L), with preserved renal and hepatic function. The remainder of the biochemical and hematological workup was unremarkable. Abdominal ultrasound identified a suprauterine ovoid pelvic mass (9 × 8 × 4.2 cm), hypoechoic peripherally with a hyperechoic center and posterior acoustic shadowing, located between the transversalis fascia and anterior parietal peritoneum.

**Figure 1.**
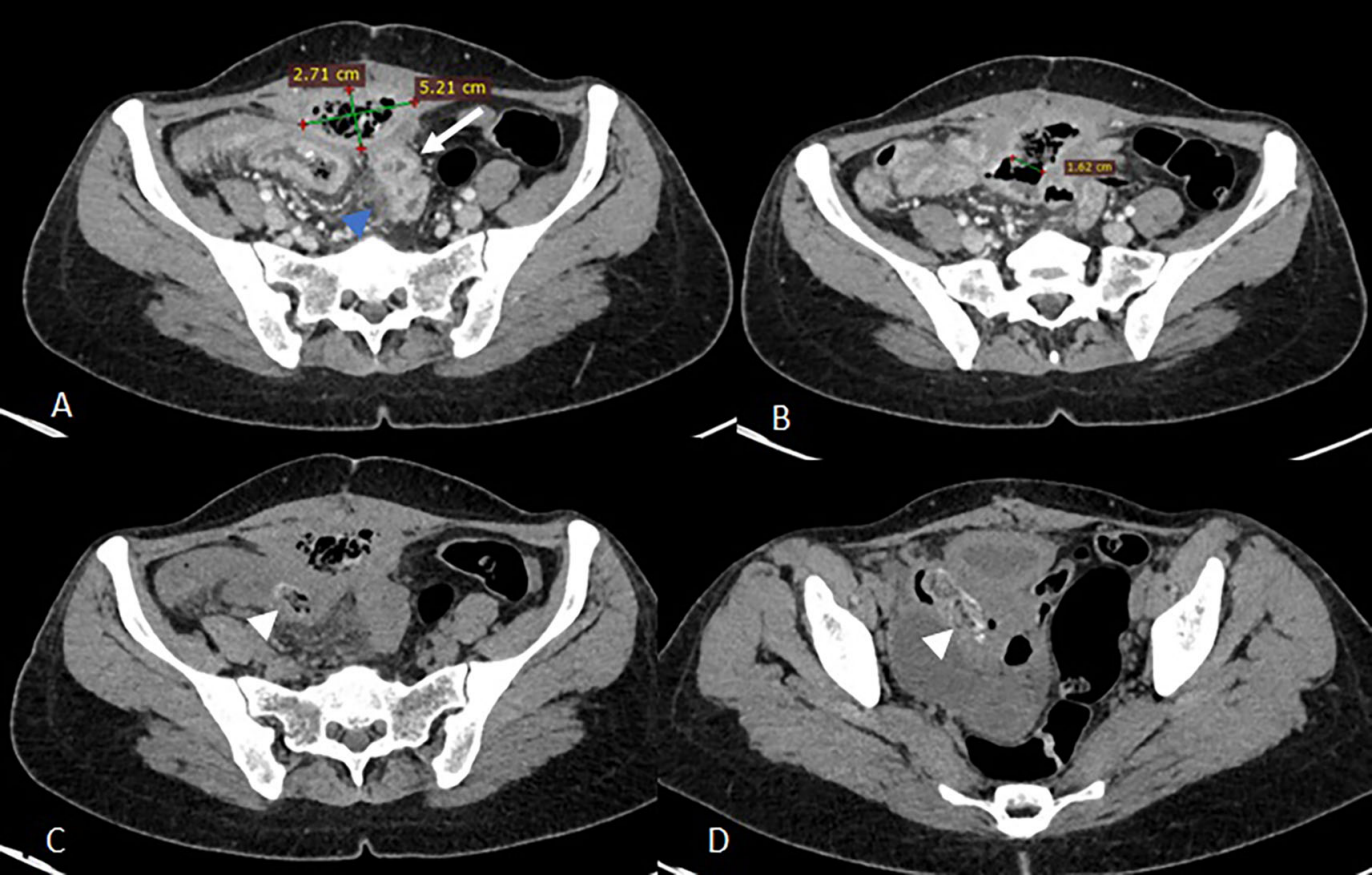
Axial scans of the abdomen and pelvis without contrast injection (images C and D) and at portal vein phase (A, B) showing a collection above the bladder in the preperitoneal space with faecal content measuring 52x27 mm (A) communicating with the lumen of an ileal loop via a 16 mm fistulous tract (B) associated with regular parietal thickening of the nearby ileal loops (arrow) and infiltration of the peritoneal fat in contact (blue arrowhead) of a reactive nature. Images C and D show two intraluminal foreign bodies in the ileal loops, spontaneously dense and heterogeneous, forming air bubbles (white arrowheads).

Abdominal computed tomography (CT) demonstrated a 9 × 8 × 5 cm pelvic-abdominal mass, anterior to the bladder and abutting the anterior abdominal wall. The lesion contained heterogeneous fluid and air with peripheral contrast enhancement, associated with agglutinated intestinal loops and multiple enlarged locoregional lymph nodes ( Figure 1). There was concern for enteric fistula and communication with the urinary bladder.

**Figure 2.**
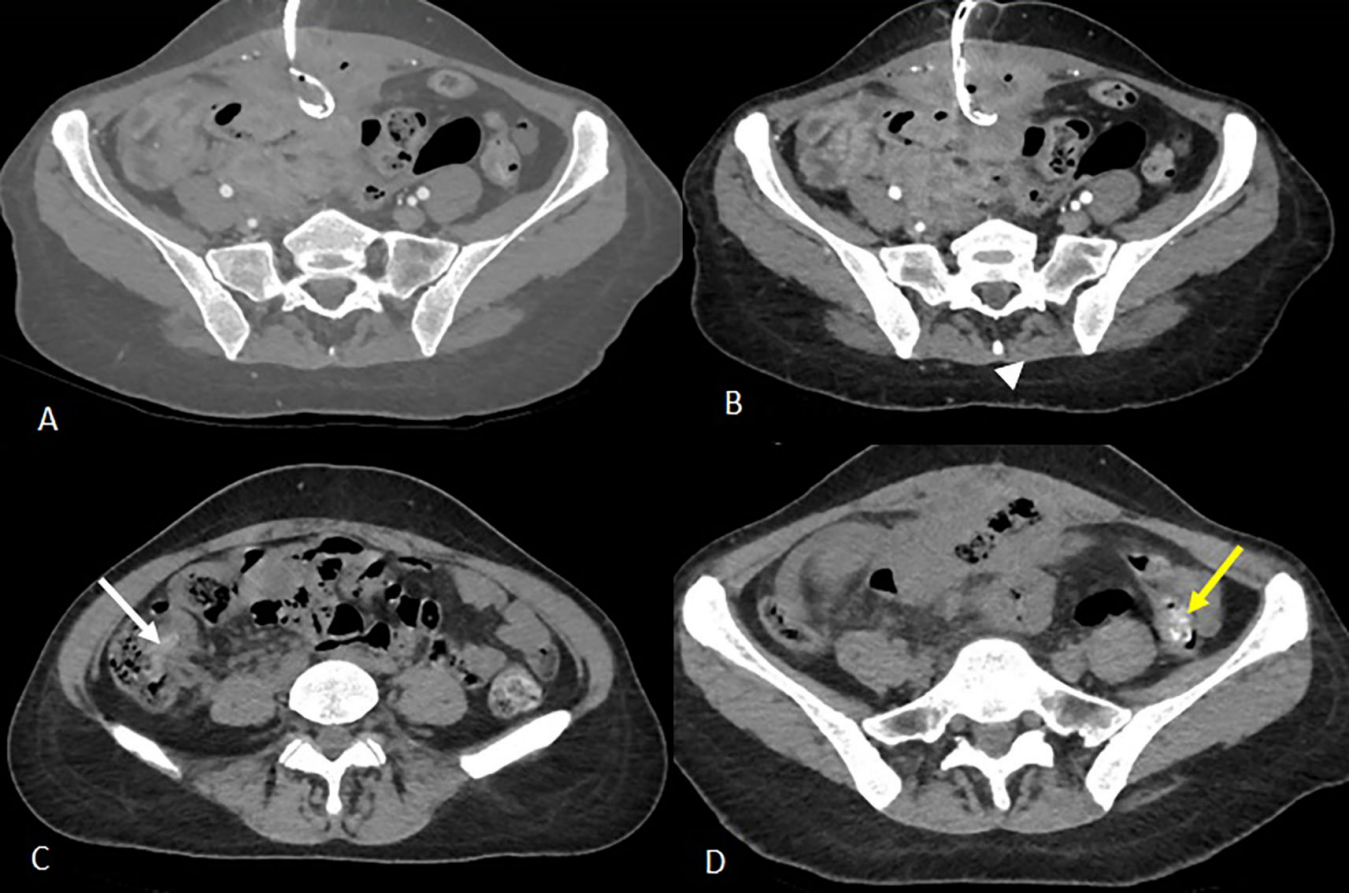
Three days after the first scan, CT-guided drainage of the collection was indicated and performed ( Figure 2). Axial slices after portal vein injection (A, B) show a drain in place with its distal end coiled in the collection (A), a stable appearance of the digestive wall thickening and the peritoneal reaction in the surrounding area previously described (B). Axial slices of the abdominal-pelvic CT scan without injection of contrast medium C and D show the migration of the two intraluminal foreign bodies from the ileal loops visualised on the previous scan. The first foreign body is found in the caecum (image C, white arrow) and the second in the left colon with its distal end in the sigmoid colon (image D, yellow arrow).

Empirical broad-spectrum antibiotic therapy with imipenem was initiated. On day 3, a radiologically guided percutaneous drainage of the collection was performed ( Figure 2), yielding a small volume of fecal material. Despite drainage, CRP rose to 140 mg/L and stabilized at 120 mg/L.

Entero-MRI revealed circumferential thickening of the terminal ileum extending over 20 cm, centered on a subparietal collection (78 × 59 × 55 mm) with a drain in place. A large fistulous communication with an adjacent intestinal loop was evident, with additional blind-ending fistulae and a spiculated border.

**Figure 3.**
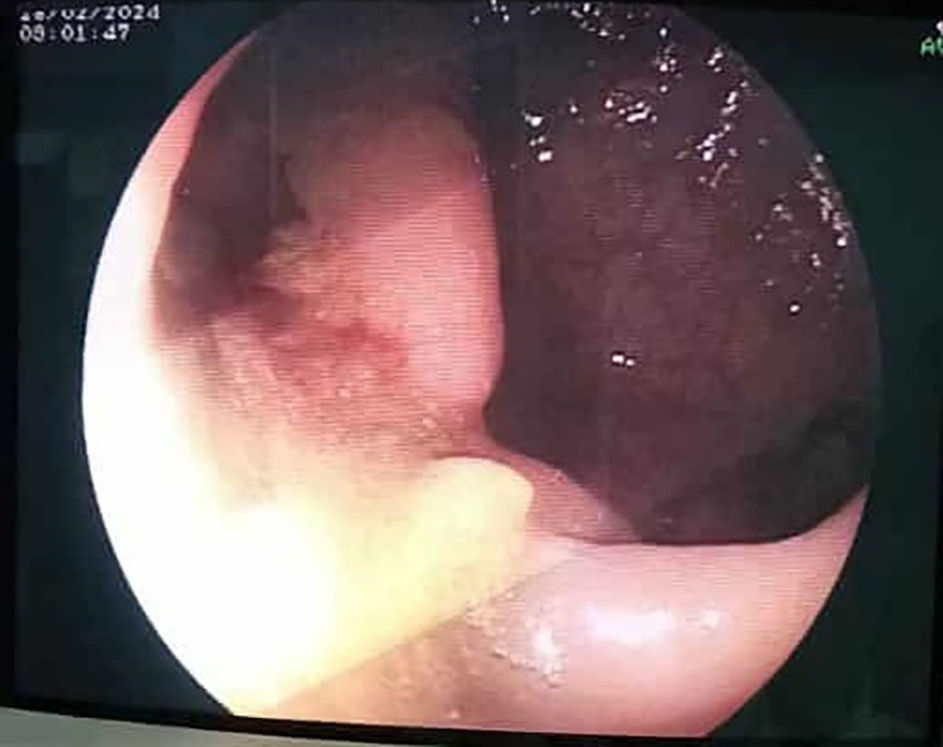
Images from the abdominal-pelvic CT scan performed 11 days after drainage: A- Axial CT scan after contrast injection at portal phase showing migration to the transverse colon of the intraluminal foreign body previously visualised at the caecal level (white arrow). There is also evidence of regular pancolic wall thickening (white arrowhead) and a stable inflammatory complex in the small bowel loops and surrounding fat. B- Sagittal reconstruction in the portal phase partially illustrating the second foreign body, which has continued to migrate to the rectosigmoid colon (previously in the left colon), the distal end of which is externalised through the anal margin (yellow arrow).

**Figure 4.**
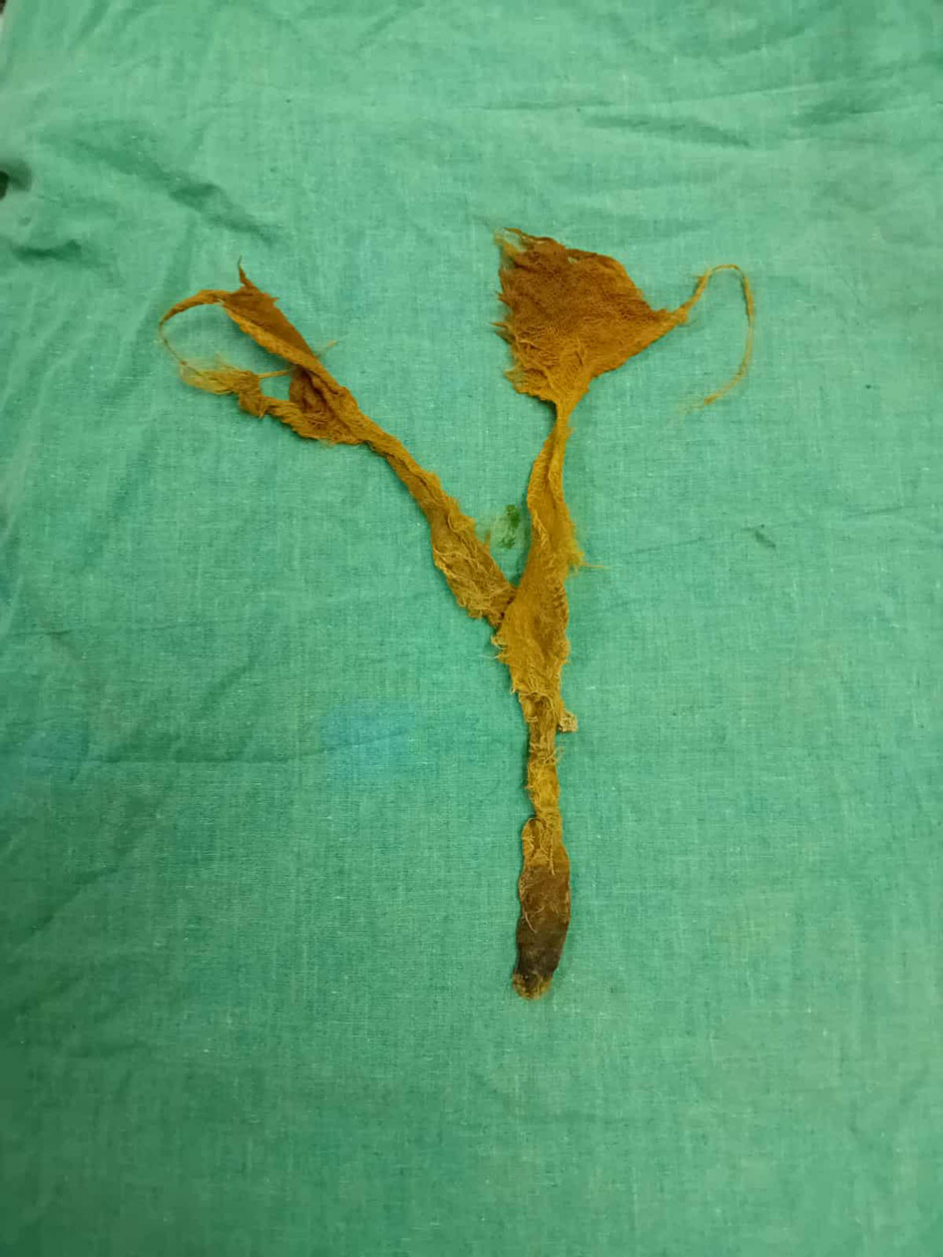
An endoscopic view of intra luminal textiloma, being externalised.

**Figure 5.**
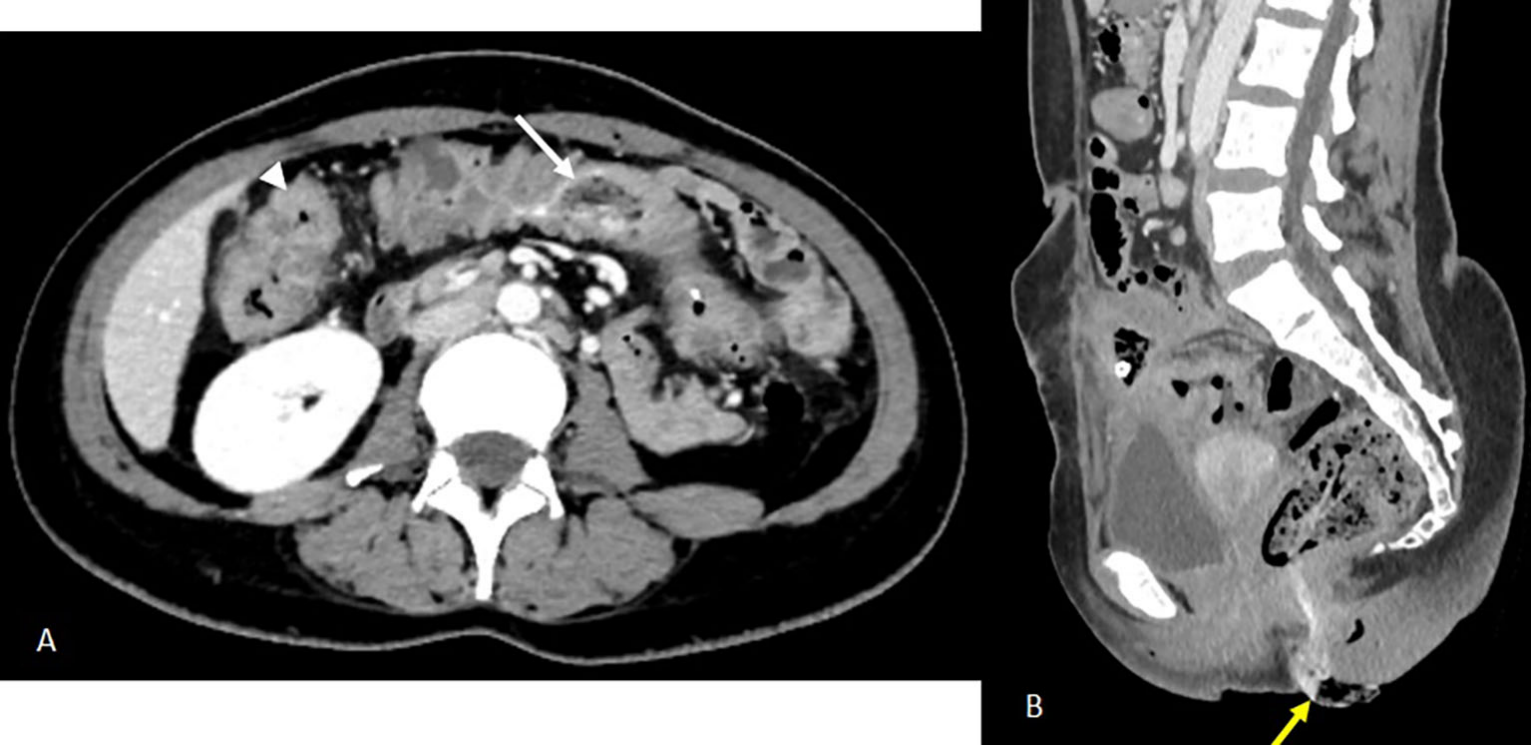
Retrieved textiloma.

On day 13, the patient reported a sensation of rectal fullness. Proctological examination revealed a textile foreign body soiled with feces. An abdominal CT scan was ordered. It confirmed the progressive migration of both foreign bodies along the colon, one reaching the transverse colon and the other advancing to the rectosigmoid with distal externalization through the anal margin ( Figure 3). Rigid rectoscopy on day 18 confirmed retained gauze embedded in the rectal mucosa approximately 20 cm from the anal verge ( Figure 4), which was extracted under visual guidance ( Figure 5). Following extraction, the patient developed acute diffuse abdominal pain and guarding.

**Figure 6.**
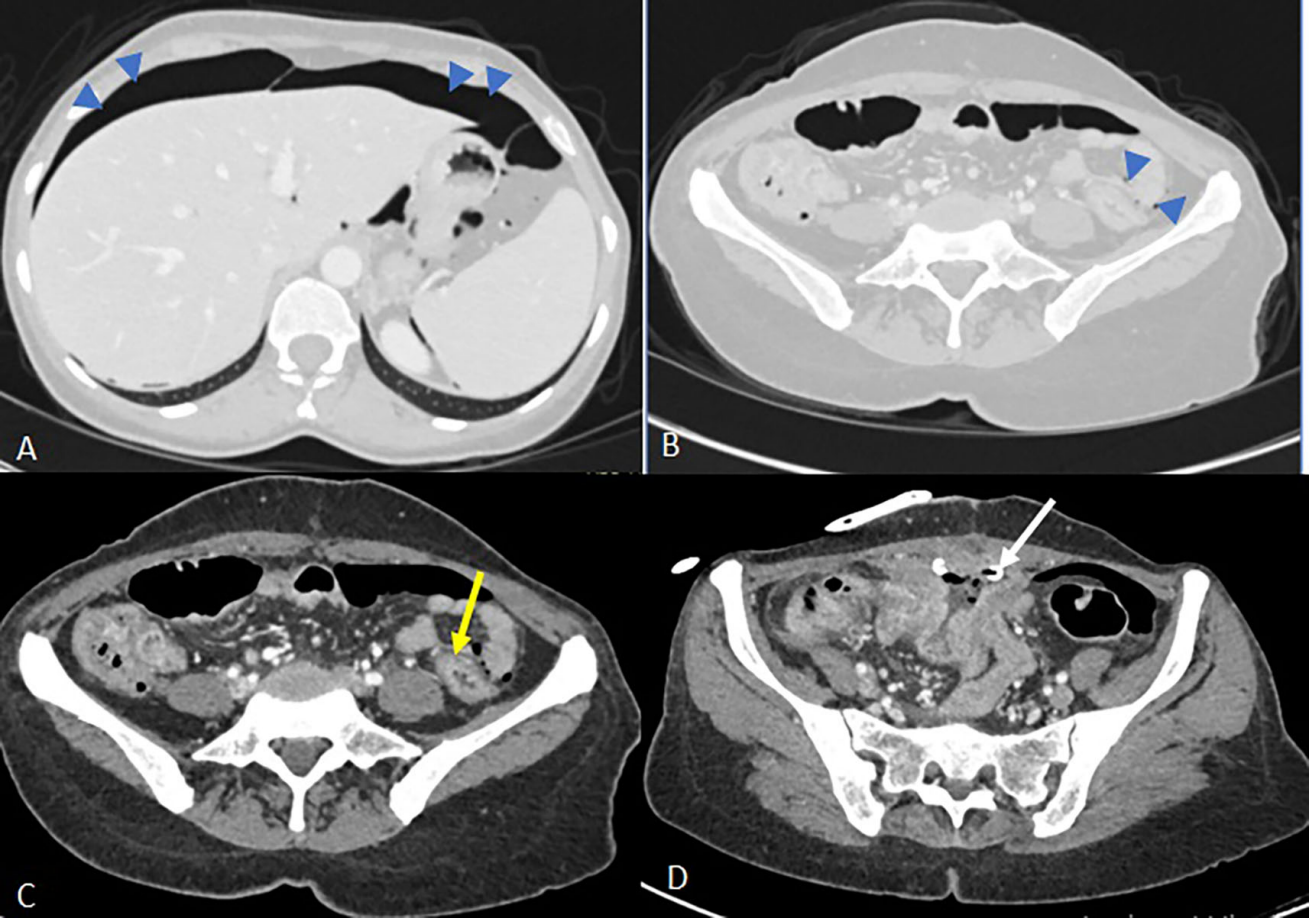
Axial images from the last scan performed approximately 3 weeks after the first scan showing: In the pulmonary window (A and B), moderate pneumoperitoneum above and below the mesocolon (blue arrowheads). Image C shows a regular circumferential thickening of the rectosigmoid junction (yellow arrow) silhouetted by extra-digestive air bubbles without any formally identifiable parietal defect. In image D, there is a partial regression of the supravesical collection with the drainage catheter in place (white arrow). However, there is persistent regular thickening of the ileal loops where the fistulous tracts are located and reactive infiltration of the surrounding fat, with no detectable intra-luminal foreign body in the digestive tract.

Emergency CT imaging revealed pneumoperitoneum, circumferential rectosigmoid wall thickening with submucosal edema, suspected parietal defect (6 cm), increased supravesical collection size, and new peritoneal fluid ( Figure 6).

After brief resuscitation, the patient underwent emergency laparotomy. A textiloma was identified, causing dense adhesions and partial intraluminal migration into an ileal segment located 230 cm from the ligament of Treitz. A second intraluminal textiloma was palpated at the right colonic angle. Multiple intestinal perforations were noted: one in the ascending colon, one in the transverse colon, two in the sigmoid colon, and three in the rectum.

Surgical management included resection of approximately one meter of small intestine with the creation of a double-barrel ileostomy, colorectal resection with a Bouilly-Wolkman double stoma, and colotomy for extraction of the second textiloma. Intra-abdominal drains were placed.

Given the patient’s hemodynamic instability and septic shock requiring high-dose norepinephrine, primary anastomoses were deferred. Despite maximal supportive care, the patient died on the first postoperative day due to multiorgan failure.

## Discussion

Gossypiboma is derived from the Latin word *gossypium* (meaning “cotton”) and the Swahili word *boma* (meaning “concealment”). ^5^ It is defined as a retained surgical sponge or gauze left in the patient’s body during a surgical procedure. These are most commonly found in the abdomen or pelvis. ^6^

This complication can affect any surgeon, regardless of experience. A recent report in gynecological departments showed that the mean age of the surgeon performing the procedure was 48 years (range: 35–59), while the mean age of the surgical nurse was 50.2 years (range: 43–56). The mean experience of the assisting nurse was 24.5 years (range: 6–36), and the mean age was 44 years (range: 30–55). ^7^

Due to the legal implications, retained surgical sponges are likely underreported, making it difficult to estimate their true incidence. ^8^ Moreover, their clinical presentation is often nonspecific or even absent. ^9^ In many cases, the complication is discovered incidentally during imaging studies, ^8^ or it presents with non-specific signs. The initial symptomatology’s inadequacy and the wide array of clinical presentations often hinder timely diagnosis. In fact, the time of declaration is often at a distance from the index operation, accounting for a median time of 12 months between the index procedure and diagnosis of retained sponges. ^10^ While computed tomography (CT) is the most accurate radiologic modality for diagnosis, with an accuracy rate of 81.8%, ^11, 12^ radiological exploration may be inconclusive due to the pleomorphic radiological spectrum. ^13^ Differential diagnosis can be challenging and may include serious affections such as abscesses, tumors, hematomas, or ruptured hydatid cysts. ^11, 14, 15^ Characteristic imaging findings on ultrasound and CT—such as a fluid collection with internal wavy structures—can help confirm the diagnosis, ^15^ but not always present. Therefore, a high index of suspicion must be maintained, particularly in high-risk situations.

A US based team recalled medical records associated with all claims or incident reports of a retained surgical objects. ^16^ They concluded that patients with retained foreign bodies were more likely than controls to have an emergency surgery (risk ratio, 8.8 [95% confidence interval, 2.4 to 31.9]), unplanned change in the operation (risk ratio, 4.1 [95%confidence interval, 1.4 to 12.4]), and body-mass index (risk ratio for each one-unit increment, 1.1 [95% confidence interval, 1.0 to 1.2]). ^16^ Another study found that associated factors with a significantly higher risk of retained foreign objects were the total number of major procedures performed [odds ratio 1.6; 95% confidence interval, 1.1-2.3; P = 0.008] and an incorrect count (odds ratio 16.2; 95% confidence interval, 1.3-197.8; P = 0.02). ^17^ Similarly, a multi-institutional study found that unexpected intraoperative events (OR: 6.97; 95% CI: 2.04–23.7; *p* = 0.002) and longer procedure duration (OR per hour: 1.41; 95% CI: 1.03–1.92; *p* = 0.032) were independent risk factors. ^18^

A detailed surgical history, including known risk factors, should always be communicated to the radiologist, as this can facilitate earlier diagnosis.

Surgical intervention is required to remove the foreign body. However, spontaneous expulsion through the rectum ^10^ or external urethra ^19^ were described. What’s more, gossypibomas have a propensity toperforate adjacent viscera, ^12^ thus necessitating prompt extraction to avoid visceral damage.

Effective strategies to prevent retained surgical bodies include systematic sponge and instrument counts before cavity and skin closure, consistent and open communication within the operating teamc utilization of electronic tracking systems such as barcode and radiofrequency identification (RFID) technology. ^20^

A recent systematic review, addressing 72 articles, conjecturedthat implementation of new technologies, such as RFID, has been shown to improve patient safety and reduce costs associated with retained soft items. Additionally, magnetic retrieval devices, sharp detectors and computer-assisted detection systems appear to be promising tools for increasing the success of recovery of metallic ones. ^21^ The implementation of these new procedures can decrease significantly the incidence of retained surgical objects as 80% of these occur with what staff believe is a correct count. ^1^

In 2021, American College of Surgeons emphasized that health care professionals bear ethical, legal, and moral responsibility to optimize patient outcomes. ^22^ Preventive measures could cut the incidence of retained objects by half, as near-miss events account for approximately 50% of cases. ^23^

## Conclusion

Diagnosing gossypiboma is often challenging, leading to delayed treatment. Increasing awareness is crucial for timely recognition. This complication results in additional costs, legal consequences, and emotional strain on the surgical team. Prevention is the most effective approach, primarily through systematic counting of surgical materials and use of metal markers to enable early and accurate detection.

## Declarations

### Ethics approval

Not required. This work is a single case report, which is generally not considered “research” requiring formal review by an ethics committee. The case describes the clinical management of one patient without any experimental intervention or deviation from standard care.

## Consent for publication

Written informed consent for publication of their clinical details and clinical images was obtained from the parent relative of the patient.

## Data Availability

*Zenodo.* CARE checklist textiloma, 10.5281/zenodo.16944366. ^24^ The project contains the following underlying data:
• CARE checklist for textiloma Data is available under the terms of the Creative Commons Zero v1.0 Universal (CC0).
