# Supplementary material for: Case Report: A Painful Twist of Fate Due to Intra-abdominal Textiloma
Source: F1000Res. 2025 Dec 4;14:947. Originally published 2025 Sep 19. [Version 2] doi: 10.12688/f1000research.169895.2 (PMC12770883; doi:10.12688/f1000research.169895.2)
Supplement: Peer review history

# Reviewer report: Reviewer response for version 2

**DOI:** 10.5256/f1000research.192324.r438580

**Published:** 2026-01-05

**Authors:** Tetsuya Ishimaru

**Affiliations:** 1 Pediatric Surgery, National Center for Child Health and Development, Setagaya, Tokyo, Japan

I’m truly sorry for the delay in my response to you. I appreciate that the authors addressed my previous comments seriously and am satisfied with the corrections. I identified two subtle issues that should be addressed.

 The second sentence of the 6th paragraph in the Discussion section, “toperforate” should be separated with a space.

 The first sentence of the 8th paragraph in the Discussion section, “conjecturedthat” should be separated with a space.

Are enough details provided of any physical examination and diagnostic tests, treatment given and outcomes?

Yes

Is the case presented with sufficient detail to be useful for other practitioners?

Yes

Is sufficient discussion included of the importance of the findings and their relevance to future understanding of disease processes, diagnosis or treatment?

Yes

Is the background of the case’s history and progression described in sufficient detail?

Yes

Reviewer Expertise:

Pediatric surgery

I confirm that I have read this submission and believe that I have an appropriate level of expertise to confirm that it is of an acceptable scientific standard.

# Reviewer report: Reviewer response for version 1

**DOI:** 10.5256/f1000research.187284.r430364

**Published:** 2025-11-25

**Authors:** Tetsuya Ishimaru

**Affiliations:** 1 Pediatric Surgery, National Center for Child Health and Development, Setagaya, Tokyo, Japan

This is a case report of a young woman who, unfortunately, died of an iatrogenic complication, gossypiboma. Sharing this tragedy and the insights learned from this case is necessary to prevent such a disaster from happening again. I appreciate the authors’ courage in revealing this case, but there are some concerns that need to be addressed.

 (Major)

 I agree that this article is not a study but a case report, and understand that the authors got approval for publication of the case from the bereavement. However, it seems better for the authors to inform the ethics committee at your institute about publishing this case in the journal to avoid any future possible conflicts, as the outcome of the case was unfortunate.

 (Minor)

 The images in Figures 3, 4, and 5 do not align with their explanations. Please correct them.

 In hindsight, can the author identify the artificial foreign bodies on the CT images performed on 11 days after drainage?

 It’s unclear when the last emergent contrast CT scan was performed. Please clarify it.

Are enough details provided of any physical examination and diagnostic tests, treatment given and outcomes?

Yes

Is the case presented with sufficient detail to be useful for other practitioners?

Yes

Is sufficient discussion included of the importance of the findings and their relevance to future understanding of disease processes, diagnosis or treatment?

Yes

Is the background of the case’s history and progression described in sufficient detail?

Yes

Reviewer Expertise:

Pediatric surgery

I confirm that I have read this submission and believe that I have an appropriate level of expertise to confirm that it is of an acceptable scientific standard, however I have significant reservations, as outlined above.

# Reviewer report: Reviewer response for version 1

**DOI:** 10.5256/f1000research.187284.r430359

**Published:** 2025-11-20

**Authors:** Muhammad Nabeel Saddique

**Affiliations:** 1 King Edward Medical University, Lahore, Pakistan

This case report describes a rare and severe presentation of intra-abdominal textiloma occurring 15 years after open ovarian cystectomy. The topic is clinically significant, as retained surgical items remain a preventable cause of morbidity, mortality, and medicolegal consequence. The authors present a thorough narrative of the case, including imaging, operative details, and clinical outcome. The report adheres to CARE guidelines and provides visual documentation that enhances understanding. However, several aspects could be improved to strengthen clarity and impact. The clinical course should be presented in a clearer chronological sequence, ideally summarized in a short timeline or table. Imaging findings could be condensed to highlight key diagnostic clues and to clarify why the retained sponge was not suspected earlier. The discussion, while comprehensive, reads more as a literature summary than an analysis; it would benefit from emphasizing the unique aspects of this case—dual sponge migration, delayed presentation, and fatal postoperative course—and from expanding on system-level preventive strategies such as surgical count protocols, barcode or RFID tagging, and team communication. Language and phrasing should be refined for academic tone and fluency, as several expressions are informal. References should be standardized and updated with recent data on global incidence and safety measures. This is a valuable and instructive report on a critical patient safety issue. With minor revisions to structure, discussion depth, and language, it will make a meaningful contribution to surgical literature.

Are enough details provided of any physical examination and diagnostic tests, treatment given and outcomes?

Yes

Is the case presented with sufficient detail to be useful for other practitioners?

Yes

Is sufficient discussion included of the importance of the findings and their relevance to future understanding of disease processes, diagnosis or treatment?

Yes

Is the background of the case’s history and progression described in sufficient detail?

Yes

Reviewer Expertise:

Meta-analyses, Systematic and Narrative Reviews, and Clinical Case Reports in the fields of Cardiology, Surgery, and Oncology, with an emphasis on evidence synthesis and clinical outcomes research.

I confirm that I have read this submission and believe that I have an appropriate level of expertise to confirm that it is of an acceptable scientific standard.

# Reviewer report: Reviewer response for version 1

**DOI:** 10.5256/f1000research.187284.r416483

**Published:** 2025-10-07

**Authors:** Faiz Tuma

**Affiliations:** 1 Central Michigan University College of Medicine, Saginaw, Michigan, USA

The article discusses interesting case but not uncommon. There are 22 authors for this case report. This is a very large number for a case report. The case is poorly written with numerous contextual mistakes. Here are some examples of the mistakes and deficiencies:

 - "clinical examination revealed a patient with altered general condition (ECOG Performance Status = 2)" - No clear what that means.

 - "centered on a subparietal collection

 with additional blind-ending fistulae and a spiculated border" - Unclear.

 "On day 13" -  what was the management during the 13 days?

 - "Proctological examination"  - what does this exactly mean?

 - "It confirmed the progressive migration of both foreign bodies" – two FBs found?

 - "the other advancing to the rectosigmoid with distal externalization through the anal margin ( Figure 3)" – not clear what that means.

 - "Figure 3. Images from the abdominal-pelvic CT scan performed 11 days after drainage: A- Axial CT scan after contrast injection at portal phase showing migration to the transverse colon of the intraluminal foreign body previously visualised at the caecal level (white arrow)" – it is not CT. It’s proctoscopy image.

 - "Figure 4. An endoscopic view of intra luminal textiloma, being externalized" – Not endoscopic view. Its specimen picture.

 - "Another study found that associated factors with a significantly higher risk of retained foreign objects were the total number of major procedures"  - no clear what this means?

 - "such as RFID" – not clear what RFID is

Are enough details provided of any physical examination and diagnostic tests, treatment given and outcomes?

No

Is the case presented with sufficient detail to be useful for other practitioners?

No

Is sufficient discussion included of the importance of the findings and their relevance to future understanding of disease processes, diagnosis or treatment?

Partly

Is the background of the case’s history and progression described in sufficient detail?

No

Reviewer Expertise:

Surgical Education

I confirm that I have read this submission and believe that I have an appropriate level of expertise to state that I do not consider it to be of an acceptable scientific standard, for reasons outlined above.
